# Hydrogels for Cardiac Restorative Support: Relevance of Gelation Mechanisms for Prospective Clinical Use

**DOI:** 10.1007/s11897-023-00630-0

**Published:** 2023-10-09

**Authors:** Valentine C. Vetter, Carlijn V. C. Bouten, Atze van der Pol

**Affiliations:** 1 Department of Biomedical Engineering, Eindhoven University of Technology, Eindhoven, The Netherlands; 2 Institute for Complex Molecular Systems, Eindhoven University of Technology, Eindhoven, The Netherlands

**Keywords:** Hydrogels, Gelation, Cross-linking mechanism, Myocardial infarction, Cardiac repair

## Abstract

**Purpose of Review:**

Cardiac tissue regenerative strategies have gained much traction over the years, in particular those utilizing hydrogels. With our review, and with special focus on supporting post-myocardial infarcted tissue, we aim to provide insights in determining crucial design considerations of a hydrogel and the implications these could have for future clinical use.

**Recent Findings:**

To date, two hydrogel delivery strategies are being explored, cardiac injection or patch, to treat myocardial infarction. Recent advances have demonstrated that the mechanism by which a hydrogel is gelated (i.e., physically or chemically cross-linked) not only impacts the biocompatibility, mechanical properties, and chemical structure, but also the route of delivery of the hydrogel and thus its effect on cardiac repair.

**Summary:**

With regard to cardiac regeneration, various hydrogels have been developed with the ability to function as a delivery system for therapeutic strategies (e.g., drug and stem cells treatments), as well as a scaffold to guide cardiac tissue regeneration following myocardial infarction. However, these developments remain within the experimental and pre-clinical realm and have yet to transition towards the clinical setting.

## Introduction

Heart failure, resulting from a myocardial infarction (MI), is associated with a high mortality and morbidity rate [1]. MI is characterized by an acute or gradual loss of functional contractile tissue, which is followed by adverse cardiac remodeling. To regenerate and/or restore the damaged myocardium with functional tissue, clinical and biomedical scientists have focused on cardiac regenerative strategies.

The discovery of culturing stem cells (i.e., bone-marrow stem cells, embryonic stem cells, and later on induced pluripotent stem cell), provided high hopes that science had found the silver bullet to cure degenerative diseases, including MI [2]. Decades of research were invested to regenerate the heart by means of the administration of stem cells, from the experimental all the way to the clinical setting, with very limited effect [3, 4]. For instance, the direct injection of stem cells suffered from low cell survival and retention within the injured myocardium [5, 6]. To circumvent this, more recent stem cell-based regenerative approaches have been developed in which the stem cell or other therapeutic agents (i.e., drugs or growth factors) are encapsulated within biomaterials, such as hydrogels, which has greatly improved cell retention, survival, and provides cells protection from the host immune system [7–9]. More importantly, hydrogels by themselves have also been shown to stimulate cardiac repair, by providing a beneficial microenvironment for cell infiltration, proliferation, and differentiation, or by supplying mechanical support to the damaged myocardium while maintaining its shape and function throughout the cardiac cycle [7, 10].

Although hydrogels can come in many flavors, one key aspect which greatly influences its application as a therapeutic agent in regenerative medicine, particularly in the cardiac setting, is related to the method of gelation of hydrogels. There are two main classes of hydrogel gelation mechanisms, physically cross-linked and chemically cross-linked. The gelation mechanism not only influences the biocompatibility, biodegradability and tunability of the hydrogel, but it also effects the method of delivery [11]. Based on the gelation method employed, there are two main strategies for the delivery of hydrogels; 1) in the form of an injectable hydrogel which transitions from a liquid to a solid state when injected within the body, and 2) in the form of a hydrogel patch whereby the hydrogel is solidified prior to transplantation onto the cardiac wall. Both injectable and patch hydrogels can form the basis for strategies aimed at tissue regeneration, since they are ideally suited as carrier systems for additional therapeutic agents (e.g., cells, cytokines, growth factors, etc.), as well as providing a regenerative micro-environment on its own.

In this review, we summarize the current advances in hydrogel technology with a particular focus on the most commonly applied gelation methods of hydrogels for cardiac application in the experimental and pre-clinical setting. Thereby setting the stage for follow-up studies aimed at developing hydrogel-based strategies targeting cardiac regeneration in the translational setting.

## What Is a Hydrogel?

Hydrogels are three-dimensional networks of hydrophilic polymer chains that can absorb large amounts of water or biological fluids [12, 13]. Hydrogels can be derived from either natural or synthetic polymers. Natural hydrogels can be from human, animal, or plant-based origins. These natural hydrogels can be either generated from a single polymer source (i.e., collagen, gelatin, agarose, hyaluronic acid, chitosan, etc.) or can be directly derived from natural tissues. The latter are obtained by means of decellularizing tissue, leaving behind only the extracellular matrix (ECM), which can then be utilized to generate a hydrogel [14•]. An advantage of a hydrogel based on decellularized ECM is that the gel preserves most of the native environment of cells, supporting their potential regenerative capacity [15]. By mimicking the native environment, naturally occurring hydrogels further provide excellent biocompatibility, also because their degradation products consist of naturally occurring substances. These hydrogels can be fabricated based on ECM components of the targeted tissue microenvironment, however their mechanical properties (i.e., rigidity and stretchability) are poor [16–19]. Additionally, being biologically derived these hydrogels show batch-to-batch variations, can elicit and immune response and there is a chance of pathogen transmission[20]. In contrast, owing to their synthetic nature, synthetic hydrogels (i.e., PEG derivatives, polycaprolactone, polyvinyl alcohol, ureido-pyrimidinone, etc.) do not suffer from batch-to-batch variation, and do not elicit an immune response or transmission of pathogens. Additionally, these synthetic hydrogels have relatively good mechanical properties, withstanding strong mechanical loads, although they suffer from poor biological activity and biocompatibility [19]. Therefore, synthetic hydrogels are in need of additional biofunctionalization to guide cell differentiation and proliferation. Changing the chemical or physical structure of the polymer would result in different mechanical properties including degradation rate, gelation time, stiffness of the gel and could also influence its biocompatibility. As an alternative, hybrid hydrogels combine the properties of both natural and synthetic polymers, including high tunability and favorable biocompatibility. However, the exact mechanism of how these hybrid hydrogels function in an in vivo setting has yet to be investigated.

## Gelation Mechanisms of Hydrogel Networks

**Fig. 1:**
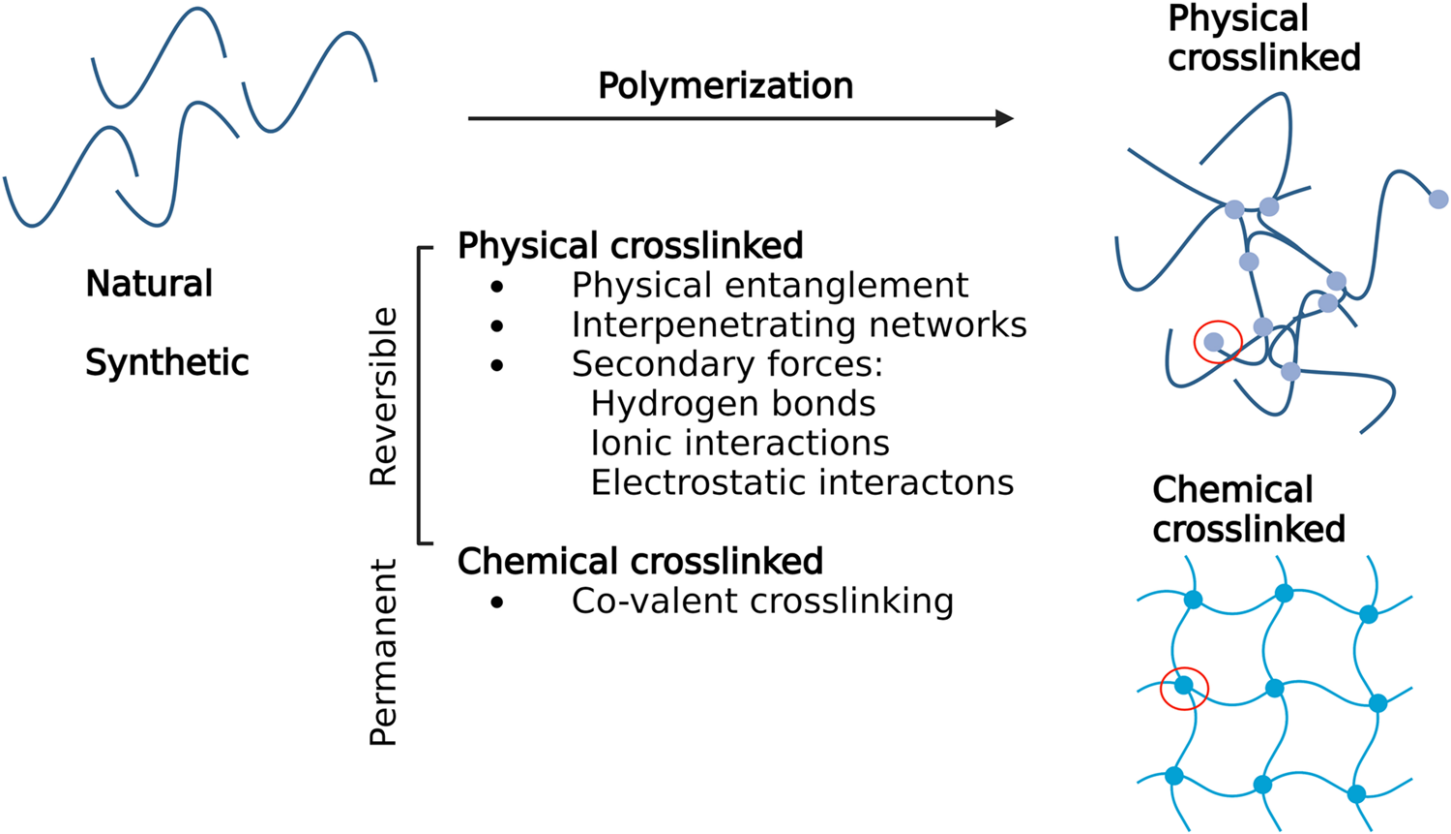
Schematic overview of gelation mechanisms of a polymeric network. A natural or synthetic polymer (**A**) can form a network via physical or chemical cross-linking (**B**), resulting in a reversible network for physical cross-linked gels and a permanent network for chemical cross-linked gels

One of the most important characteristics of hydrogels is their gelation, which is marked by the transition from a liquid solution to a solid gel (sol–gel transition). In a hydrogel, this sol–gel transition can take place through either chemical or physical cross-linking (Fig. 1) [21, 22].

Physically cross-linked hydrogels contain reversible links that can be created by non-covalent reversible interactions (via physical entanglement, interpenetrating networks, or secondary forces). This system is highly tunable. The degradation time can be tailored by the precursor molecules, while stiffness and gelation time can be adjusted by the percentage of precursors in solution. However, due to the reversible nature of these cross-links, physical interactions can easily be broken, resulting in mechanically weaker gel properties and relatively fast degradation rates. On the other hand, chemically cross-linked hydrogels form through the formation of covalent bonds. They have the advantage of being very controllable, mechanically robust, and highly stable. However, they often use potentially toxic reaction initiators and can release free radicals during the gelation process, rendering them less suitable for in vivo use.

### Physical Cross-linked Hydrogels

**Fig. 2:**
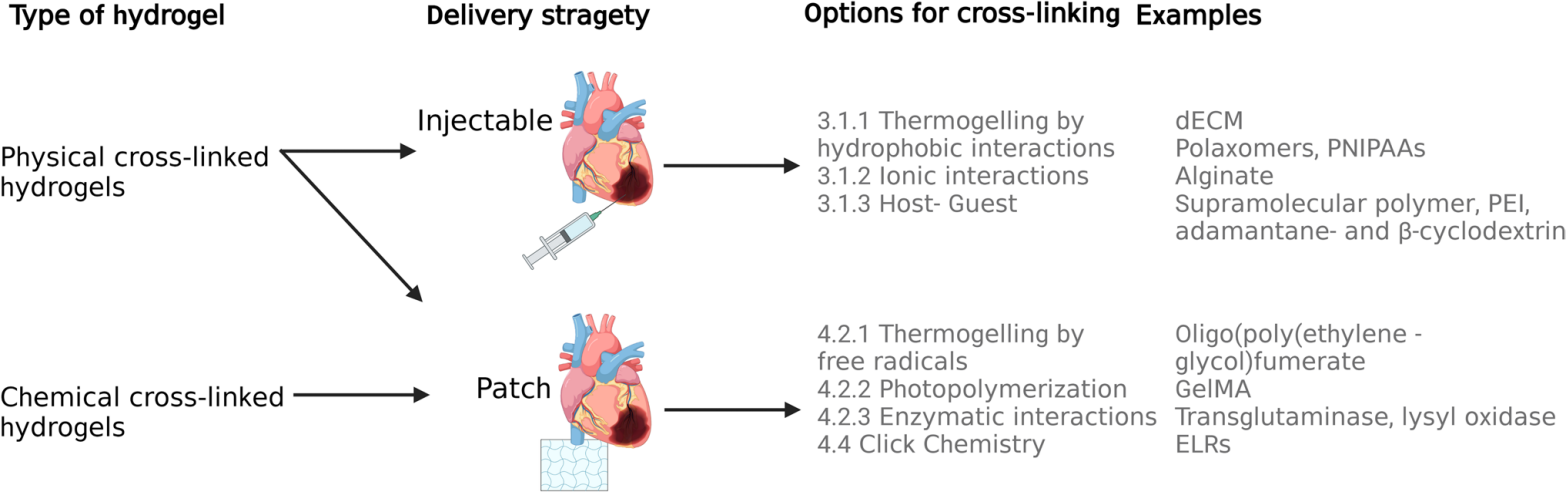
Schematic overview illustrating how the choice of hydrogel gelation mechanism influences the route of administration and, consequently, their cardiac application in vivo

Physical cross-linking of hydrogels results in reversible links that can self-assemble into a hydrogel when mixed. Most physically cross-linked hydrogels are driven by reversible self-assembly, which is dependent on opposite charge interactions. The resulting cross-linking can take place via amphiphilic copolymers, hydrogen bonds, ionic interactions, crystallization, protein and host–guest interactions [21–23]. In the next section we concentrate on the main hydrogel cross-linking mechanisms that are relevant and suitable for administration by means of injection within the cardiac setting (Fig. 2).

#### Thermogelling Through Hydrophobic Interactions

Thermogelling is a process by which temperature-responsive hydrogels can form a sol–gel transition through changes in temperature by physical cross-links, independent of a chemical reaction [24]. In this case, the formation of a hydrogel is driven by hydrophobic interactions between polymers. These hydrophobic polymers are soluble when maintained at a low temperature, and as soon as the temperature is increased, the polymers become more hydrophobic, leading to an increase in hydrogen bonds between the polymers resulting in the generation of polymer aggregates [25]. All polymers containing a hydrophobic domain, like amphiphiles, can be used to form a thermogelling hydrogel. A typical characteristic of these gels is that hydrogen bonds are relatively weak and therefore these gels have low mechanical strength and a fast degradation rate. Decellularized ECM hydrogels are a perfect example of a natural hydrogel that utilizes thermogelling-based cross-linking [15, 26, 27]. The synthetic based poloxamers and poly(*N*-isopropylacrylamide) (PNIPAAm) hybrid synthetic hydrogels are also able to undergo gelation by means of thermogelling. Noteworthy is that the poloxamer hydrogels have hemorheological and anti-thrombotic properties, making them ideal candidates for implementation in cardiac regenerative strategies. Poloxamers and PNIPAAm synthetic based hybrid hydrogels have been shown to be effective in the pre-clinical setting in treating strokes, MI and ischemia–reperfusion injuries [28, 29]. Additionally, a recent thermogelling synthetic hydrogel (poly(NIPAAm-co-HEMA-co-MAPLA)) has been tested as a therapeutic strategy in a rat and a porcine model for MI [30]. Interestingly, in both studies the synthetic hydrogel led to a reduction in myocardial remodeling as well as an improved cardiac function following MI.

#### Ionic Interactions

Hydrogels cross-linked by ionic interactions can take advantage of the physiological internal pH value of the human body. These pH-dependent gels undergo a sol–gel transition as a result in changes in pH values [22, 24]. This sol–gel transition is driven by the interaction between anionic and cationic molecules, which, due to their opposing charges, form ionic complexes. Ionic hydrogels show good biocompatibility with a gelation time from seconds to minutes, which can be adjusted by the molecular weight, composition and concentration of the precursor anionic and cationic solutions [31]. A prime example of such a hydrogel is anionic alginate cross-linked with cationic ions (i.e., calcium, sodium). Besides being a naturally derived polysaccharide and therefore possessing good biocompatibility and biodegradability, alginate also has anti-thrombogenic properties, rendering it of particular interest for cardiac applications [32]. In a porcine model for MI, treatment with an alginate hydrogel has shown to promote healing without inducing an immune response or thrombosis [33]. However, it can be challenging to adjust the gel to a defined pH value, in large part due to the variety of pH values present in the human body. Specifically, a pH change associated with an inflammation response, such as during a MI, could result the dissociation of the gel [34].

#### Host–Guest Interactions

Host–guest interactions are non-covalent bonds, including hydrogen bonds, hydrophobic and electrostatic interactions, that can self-cross-link in a complementary and reversible manner between two molecules [35]. This cross-linking mechanism is ideally suited for hydrogels injected into the myocardium due to being a highly selective reaction, and therefore limited interference to the gelation process can be expected from cardiac tissue components. Furthermore, these host–guest interactions can occur under physiological conditions, while also being highly tunable. In addition, by combining multiple host–guest interactions into a single hydrogel, the strength and durability of the hydrogels can be significantly increased [36]. Hydrogels from supramolecular polymers are a prime example of host–guest hydrogels that take advantage of multiple interactions; so called orthogonal non-covalent interactions [22]. These polymers possess great tunability and high specificity. One such supramolecular polymer-based hydrogel, was designed utilizing host–guest interactions between acrylated β-cyclodextrin (Ac-β-CD) monomers and the aromatic residues, phenylalanine, tyrosine and tryptophan, of gelatin [37]. This specific combination of supramolecular polymers can be photo cross-linked by UV-light, resulting in a hydrogel formed of both weak host–guest interactions and stronger co-valent photo-induced cross-links. The advantage of these two interaction mechanisms is that the resulting hydrogel is mechanically strong, yet maintains its ability to self-cross-link upon disruption by subsequently re-assembling the host–guest interactions. As another example, host–guest interactions have been used to develop a hyaluronic acid (HA) based hydrogel modified with adamantane- and β-cyclodextrin as a delivery system for endothelial progenitor cells in a rat model for MI [38]. This host–guest interaction-based HA hydrogel promoted endothelial progenitor cell survival and improved ventricular function and reduce remodeling following MI. A similar approach has been developed for cardiac repair using a polyethylenimine (PEI) and polyethylene glycol host–guest interaction synthetic hydrogel, which has been shown to be effective in the delivery of a siRNA treatment into the rat myocardium [39]. However, the effect on cardiac repair of this hydrogel remains to be explored.

### Chemical Cross-linked Hydrogels

A second class of hydrogels revolves around chemical cross-linking of hydrogels. There are multiple types of chemically cross-linked hydrogels, including through radical polymerization, high energy irradiation, enzymes and complementary groups [21]. For cardiac repair purposes, the polymerization reaction of these hydrogels is often thermally or photo-initiated. For both reactions, the sol–gel transitions are driven by an initiator, creating a free radical that in turn starts the polymerization or cross-linking reaction [9, 22]. Besides the use of toxic initiators for the cross-linking of gels, chemically cross-linked hydrogels can also take place through click chemistry or enzymatic reactions. The advantage of chemically cross-linked hydrogels is that the co-valent bonds result in mechanical stronger gels, with a slower degradation time. Below, we concentrate on the hydrogel cross-linking mechanisms that have so far proven suitable for the administration to the heart by cardiac patch formation (Fig. 2).

#### Thermogelling Through Free Radical Polymerization

Thermogellation by means of radical polymerization takes advantage of changes in temperature to induce the sol–gel transition. However, unlike thermogellation by hydrophobic interactions, these systems take advantage of free radicals as reaction initiators. One limitation of the thermogelling hydrogels is that they undergo shrinkage upon temperature increase [40]. In a murine MI model, an oligo(poly(ethyleneglycol)fumarate) hydrogel was combined with graphene-based nanoparticles to create a conductive injectable hydrogel. Four weeks following the injection into the post-MI myocardium, a reduction in infarct size coupled to an improvement in left ventricular ejection fraction was observed, when compared to sham treated animals [41]. It must be noted, that this study utilized a thermogelling strategy which could have negative effects when injected into the myocardium due to the use of toxic free radicals as gelation initiator. Especially considering the prolonged gelation time described (above 20 min). Ideally the preferred route of delivery for this gelation mechanism would be a cardiac patch-based hydrogel.

#### Photopolymerization Through Free Radical Polymerization

For photo-initiated systems, the chemical sol–gel transition is initiated by light [42]. The advantage of this mechanism is that the transition to a high viscous solution occurs within seconds to minutes, and the minimum amount of heat released during this reaction ensures its compatibility for cells and drug delivery. Furthermore, the number of cross-links is regulated by the exposure to (UV) light, directly influencing properties like stiffness and degradation time of the hydrogel. It has been observed that this type of cross-linking is less suitable for gelation in vivo since the propagation of light closely relates to the optical properties of tissue, showing a large variation in the presence of fat or blood [43]. If a light source is applied external to the body, this could lead to an uneven light exposure and therefore an uneven cross-linking of the hydrogel in vivo. The most well-known photo cross-linkable hydrogel is the hybrid gelatin methacrylate (GelMA) system. Gelatin is denatured collagen, one of the prominent components of the cardiac ECM. It contains an arginine-glycine-aspartic acid peptide sequence to which cells can easily attach, and the hydrogel can be remodeled by cells due to the presence of a matrix metalloproteinase sequence. The mechanical and physical (pore size, degradation rate) properties of GelMA hydrogels are easily tunable, and can incorporate additional growth factors and cytokines [44]. Depending on the number of cross-links, precursor weight percentage, cell type, and remodeling activity, GelMA can be stable at 37° for weeks [45]. GelMA hydrogels have been used for a wide range of cardiac applications, including hydrogel-based cardiac patches that allow for electrical transmission and have demonstrated to promote cardiac tissue healing when injected into the infarct of murine hearts [44, 46–49].

#### Enzymatic Interactions

Chemically cross-linked gels that do not require the use of initiators and free radicals are enzymatically cross-linked gels. Cross-linking by enzymes still results in the formation of covalent bonds, but in a substrate-specific manner [50]. The greatest benefit of this mechanism lies in the use of enzymatic catalysis, which is a naturally occurring process within the human body. After exposure of the enzyme these hydrogels undergo a relatively fast cross-linking reaction. Although few enzymatically cross-linked hydrogels have been explored for cardiac regeneration, there are various enzymes that could serve as a catalyst including transglutaminase and lysyl oxidase (LO). Transglutaminase is a calcium-dependent enzyme, involved in wound healing and this enzyme is upregulated upon MI. Transglutaminase catalyzes the formation of covalent bonds between lysine and glutamine residues, resulting in the formation of cross-links within 2 min of mixing the enzyme with its substrates [51, 52]. One example is a self-assembling injectable hydrogel that was mechanically reinforced by the introduction of a transglutaminase mediated co-valent cross-link. The hydrogel was used to encapsulate salvianolic acid B, and upon injection into the myocardium of a murine MI model, this gel remained present for at least 20 days in the ventricular wall of the murine heart and was shown to improve cardiac function [53•]. Other enzymes involved in tissue remodeling following MI are LOs. LOs initiate co-valent cross-links between collagens in the infarcted region and could be a promising source to create a hydrogel for use in the heart [54]. In vitro, it was shown that a LO cross-linking peptide hydrogel seeded with human mesenchymal stem cells (MSCs) increased in mechanical strength overtime (by the number of cross-links) in the presence of active LO [55]. Following a MI, the produced LO could help to keep the hydrogel intact and support the infarcted zone while the affected area is being remodeled. In addition, excessive collagen remodeling is associated with cardiac fibrosis and heart failure [54]. A LO cross-linking hydrogel could entrap redundant LO, preventing the muscle from additional stiffening.

#### Click Chemistry

Hydrogels cross-linked via click chemistry undergo a chemical reaction whereby molecules are permanently linked, in a highly selective manner. This can occur under physiological conditions, resulting in minimal interference and side effects in vivo [56]. Typically, the formation of the cross-links in click hydrogels is highly tunable, rendering this hydrogel suitable for the application as a cardiac patch as well as an injectable hydrogel. Up till now, only a few click hydrogels have been investigated for use in MI. The most recent being a hydrogel comprised of multiple click-based reactions, whereby an initial cardiac patch-based hydrogel was attached to the infarcted area by a secondary click chemistry “glue,” forming bonds between the aldehyde groups in the patch and amino groups of the myocardium. This was followed by the supplementation of a biocompatible click chemistry hydrogel encapsulating MSCs on top of the cardiac patch, which extended the retention time and improved the cytocompatibility of the patch [57]. Furthermore, a noteworthy recent study used an injectable elastin-like recombinamers-based hydrogel (ELRs) with click chemistry sensitive to matrix metalloproteases (MMP) and cathepsin K (Cat K). Since both MMP and Cat K increase upon MI, this ELRs hydrogel can selectively form in the infarct zone when applied in an appropriate time window. In an ovine model for MI, this hydrogel was shown to reduce fibrosis, upregulated angiogenesis, and improve cardiomyocyte preservation in the border zone, when compared to sham treated animals [58•].

## Hydrogel Delivery

The mechanism of gelation of the hydrogel directly influences the delivery strategy to be employed. A physically cross-linked hydrogel can be either delivered by means of injection or cardiac patch, while a chemically cross-linked hydrogel is mainly suitable for delivery by means of a cardiac patch.

### Injectable Hydrogels

**Fig. 3:**
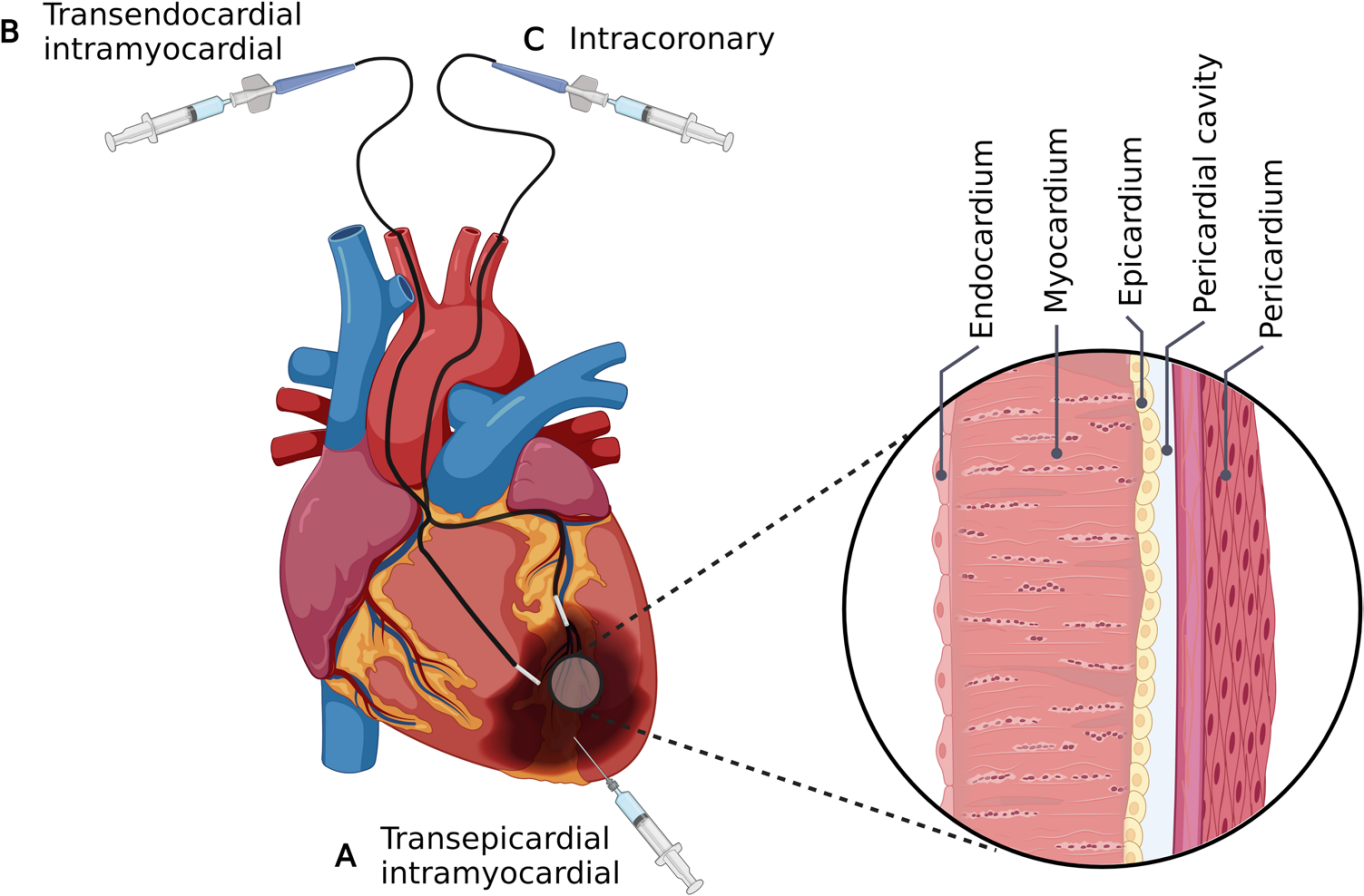
Delivery routes for injectable hydrogles via transepicardial intramyocardial injection (**A**), catheter-based transendocardial intramyocardial (**B**) and catheter-based intracoronary injection (**C**)

As previously stated, chemically cross-linked hydrogels utilize toxic reaction initiators and free radicals, which can be harmful when present in vivo, therefore the most suitable type of hydrogels for delivery by means of injection are those that take advantage of physical cross-linking for gelation. For injectable hydrogels there are currently three main strategies: transepicardial intramyocardial injection, transendocardial intramyocardial injection, and intracoronary injection (Fig. 3). Transepicardial intramyocardial injection is performed through thoracotomy and under direct visualization of the heart, allowing for precise identification of the injection site. In the pre-clinical setting, transepicardial intramyocardial injections utilizing alginate-based hydrogels, which are cross-linked by ionic interactions, have shown to improve left ventricular function and wall thickness following MI [59, 60]. Interestingly, a safety and feasibility study in patients with dilated cardiomyopathy showed no adverse effects of the treatment at 24-month follow-up [61]. Additionally, an improvement of left ventricular ejection fraction was also observed in these patients. While feasible and accurate, this approach requires a larger injection (15 injections of 0.25 to 0.35 ml) and is very invasive for the patient. The optimal course for clinical implementation of injectable hydrogels is the use of the less invasive imaging guided approach. This involves the use of imaging techniques, such as echocardiography or magnetic resonance imaging (MRI), in combination with a cardiac catheter to guide the delivery of injectable hydrogels to the heart. Imaging guidance can help to ensure accurate delivery of injectable hydrogels to the desired location, and can improve the efficacy of treatment. This is where transendocardial intramyocardial injection and intracoronary injection come into play. Transendocardial intramyocardial injection is advantageous due to its precise catheter-based navigation to the site of injury, greatly reducing the burden for the patient. One noteworthy example of transendocardial intramyocardial injection is the use of VentriGel, which is an injectable hydrogel, thermogelling by hydrophobic interactions, derived from decellularized pig hearts. This hydrogel has been shown to improve cardiac function and reduce infarct size in rats and porcine MI models [62, 63]. The first clinical study to determine the safety and feasibility of VentriGel in patients with MI and left ventricular dysfunction, demonstrated an increase in the 6-min walk test distance and decreases in NYHA functional class across the entire cohort of patients [64]. Similar to the transendocardial intramyocardial catheter-based injection approach, is the catheter-based injection through the left main coronary artery, whereby the injectable hydrogel can penetrate through the vascular wall to reach the infarcted region. Such an approach has been used to inject an alginate hydrogel (IK-5001), cross-linked by ionic interactions, in a porcine model of MI, demonstrating that the treatment improved infarct repair and prevented adverse remodeling after MI [33]. Interestingly, this has led to a clinical trial where the treatment was observed to be feasible and well tolerated within patients with moderate-to-large ST-segment–elevation Mis [65].

Overall, injectable hydrogels are promising for cardiac regenerative strategies, however, they do have several limitations. First and foremost, injectable hydrogels, in particular when applied via intracoronary injections, can cause re-embolization of the coronary artery; an extremely undesired side effect for clinical implementation [66]. Additionally, injectable hydrogels have poor mechanical strength, potential immunogenicity, and rapid clearance from the beating heart. In an effort to circumvent these safety issues, a recent pre-clinical study demonstrated a novel approach whereby the hydrogel was injected in the pericardial space [67]. This technique eliminates the risk of premature gelation and embolization, while improving hydrogel retention. Similar future advances will be essential to transition injectable hydrogel technology safely into the clinical setting.

### Hydrogel-Based Cardiac Patches

The second approach for hydrogel delivery revolves around the generation of a hydrogel patch. Here, the hydrogel is allowed to gelate ex-vivo, after which it can be transplanted onto the heart. This method increases the options of suitable gels, since both the gels suitable for injection, as well as the chemically cross-linked gels can be modified for patch delivery. To date, there are two main strategies for the implantation of a hydrogel-based cardiac patch: 1) by means of directly suturing onto the infarcted cardiac wall; by far the most common used approach, or 2) glueing the patch onto the infarcted wall [68–70]. Both large and small animal studies have demonstrated a positive effect of utilizing hydrogel-based cardiac patches with respect to cardiac function and cardiac tissue repair. However, besides being a technically difficult strategy considering the stability of an infarcted cardiac tissue, direct suturing of a hydrogel-based patch to the cardiac tissue can also elicit an inflammatory reaction or oxidative stress that could affect the regeneration process [71]. To overcome these drawbacks, researchers have opted for the utilization of biocompatible adhesives, such as fibrin glue, to secure the hydrogel-based cardiac patch to the infarcted area. This approach has demonstrated high feasibility with successful improvement in cardiac function and tissue repair [72]. Besides these two options, recent work has identified the capability of certain hydrogel-based cardiac patches to be “spray painted” on the injury in the heart. Several leading studies have demonstrated not only the effectiveness of this “spray paint” approach, in both small and large animal models for myocardial infarct, but also that this approach can improve outcome following MI [71, 73, 74]. The main benefit of a hydrogel-based cardiac patch is the reduced chance of an embolism, when compared to injectable hydrogels, however, the procedure of applying a hydrogel-based cardiac patch still requires directly visualizing the heart.

## Conclusion

While many different hydrogels are being developed and investigated, the key aspect that regulates the potential use of a hydrogel is its mechanism of gelation. In this review, we aimed to provide a comprehensive overview of the most suitable gelation methods (i.e., cross-linking mechanisms) with a specific focus on how gelation affects the use of a hydrogel for treating myocardial infarction (Table 1). After all, the mechanism of gelation determines the biocompatibility, mechanical properties, chemical structure, as well as the mode of implementation of the hydrogel.

**Table 1:** Characteristics of currently employed hydrogels for myocardial infarcts

Interaction Mechanism	Example	Animal Model	Administration Route	Gelation Time	Degradation Range	Ref
Hydrogels by chemical cross-linking						
Thermogelling	Oligo(poly(ethyleneglycol)fumarate)	Rat	Intramyocardial injection	20 Minutes	> 4 weeks	41
Photo initiated	GelMA	Rat	Patch-based (sutured)	10 Minutes	> 21 days	49
Photo initiated	GelMA	Rat	Intramyocardial injection	3 Minutes	Weeks*	48
Enzymatic	Transglutaminase-mediated	Rat	Intramyocardial injection	2 Minutes	> 20 days *(Expected up to 3 months)*	53
Click chemistry	HHA@ODS@HP-β-CD@Res	Rat	Adhesive patch-based	Seconds	28 days	57
Click chemistry	Elastin-like recombinamers-based hydrogel	Ovine	Intramyocardial injection	10 Minutes	-	58
Hydrogels by physical cross-linking						
Thermogelling	Porcine myocardial matrix	Rat	Intramyocardial injection	Minutes-1 Hour**	< 28 days**	27
Thermogelling	Poloxamer 188	Porcine	Intracoronary infusion	-	4-h follow up time	28
Thermogelling	Poly(NIPAAm-co-HEMA-co-MAPLA)	Rat/Porcine	Intramyocardial injection	Seconds	> 8 weeks *(Expected 3–5 months)*	30
Ionic interaction	Alginate	Porcine	Intracoronary infusion	Seconds-Minutes***	< 8 weeks	33
Host–guest	HA- adamantane- and β-cyclodextrin	Rat	Intramyocardial injection	Seconds-Minute	48-h follow up time *(Expected up to weeks)*	38
Host–guest	PEI and polyethylene glycol	Rat	Intramyocardial injection	< 1 Minute	> 7 days	39

GelMA = Gelatin methacrylate; HHA@ODS@HP-β-CD@Res = Hydrazided hyaluronic acid@aldehyde-dextran sponge@2-hydroxy-β-cyclodextrin@resveratrol; Poly(NIPAAm-co-HEMA-co-MAPLA) = Poly(N-isopropylacrylamide-co-2-hydroxyethyl methacrylate-co-methacrylatepolylactide; Ac-β-CD = acrylated β-cyclodextrin; HA = Hyaluronic acid; PEI = polyethylenimine

^*^Based on examples from literature^42,44–46^, **Based on examples from literature^15, 26^, ***Based on examples from literature^31,32^

It should be highlighted that the “ideal” gel does not exist and that the best choice would be application dependent. Both physically and chemically cross-linked hydrogels, administered in the form of injection or cardiac patch, to delivery cells, drugs or either by itself, have been shown to be effective in supporting and (partly) repairing the injured myocardium. That being said, the current field of hydrogel design, particularly for cardiac applications, is still in an explorative phase. To obtain a fundamental understanding of the effectiveness of the hydrogel itself and the addition of a functional component, like cells or bioactives, a systematic overview of all pre-clinical studies should be conducted to guide future translational studies.
